# Histological evaluation of different concentrations of hyaluronic-acid-added zinc oxide eugenol on rat molar pulp

**DOI:** 10.1007/s10266-024-00973-7

**Published:** 2024-07-03

**Authors:** Irmak Bektas, Afife Binnaz Hazar Yoruc, Leyla Cinel, Meltem Ekinci, Seher Eda Horoz, Deniz Mukaddes Turet, Ali Mentes

**Affiliations:** 1 Department of Pediatric Dentistry, Institute of Health Sciences, Marmara University, Basibuyuk, Saglik Yerleskesi 9/3 Maltepe, 34854 Istanbul, Turkey; 2 Department of Metallurgical and Materials Engineering, Yildiz Technical University, Davutpasa Cad. No.127 Esenler,, 34210 Istanbul, Turkey; 3 Department of Pathology, School of Medicine, Marmara University, Pendik Egitim Arastirma Hastanesi, Ust Kaynarca, Pendik, Istanbul, Turkey; 4 Experimental Animal Implementation and Research Center, Medical School, Marmara University, Basibuyuk, Maltepe, 34854 Istanbul, Turkey; 5 Department of Pediatric Dentistry, School of Dentistry, Marmara University, Basibuyuk, Saglik Yerleskesi 9/3 Maltepe, 34854 Istanbul, Turkey

**Keywords:** Dental pulp, Hyaluronic acid, In vitro/in vivo tests, Pulpotomy, Rat molars, Zinc oxide eugenol

## Abstract

Hyaluronic acid (HA), known for diverse properties, was investigated for its potential in dental pulp therapy. This study investigated the potential of HA in dental pulp therapy by examining the physical properties and effects of zinc oxide eugenol (ZOE) pulpotomy materials containing varying HA concentrations on rat molar teeth. In vitro tests assessed compressive strength and hardness of ZOE materials blended with HA (0.5%, 1%, 3%) and HA gels (0.54%, 0.8%). 120 samples, encompassing the control group, underwent compressive strength testing, while 60 samples were designated for hardness assessment. In vivo experiments on rat molars studied histological effects of HA-containing ZOE on dental pulp over 1 week and 1 month. Gels with HA concentrations of 0.5%, 1%, and 0.54% were used in pulpotomy on 22 rats. Each rat underwent the procedure on four teeth, with one tooth serving as a control, totaling 88 teeth subjected to the intervention. In the analyses, SPSS 22.0 was used and the significance level was set at *P* = 0.05. Findings showed that HA at 0.5% maintained compressive strength, but higher concentrations decreased mechanical properties significantly (*P* = 0.001). Histological assessments indicated better outcomes with lower HA concentrations in terms of odontoblast layer continuity (*P* = 0.005 at 1 month) and pulp vitality (*P* = 0.001 at 1 week and *P* = 0.018 at 1 month). The study suggests HA holds promise for pulpotomy and regenerative endodontic treatments, but further research is needed to understand long-term clinical implications.

## Introduction

Pulpotomy, a surgical procedure, entails the removal of infected or inflamed coronal pulp to safeguard the vitality and function of the remaining root pulp. It is traditionally considered an established protocol as a standard pulp therapy for primary dentition displaying indications of reversible pulpitis [1]. Preoperative pulp inflammation poses a significant consideration in clinical dentistry, particularly in cases of deep carious lesions. While vital pulp therapy (VPT), including procedures like direct pulp capping and pulpotomy, aims to maintain pulp viability and function, the impact of preoperative inflammation on treatment outcomes requires further elucidation. Recent advancements, notably the introduction of calcium silicate-based cements, have expanded the scope of VPT, with hydraulic calcium silicate cement indicating promise for managing irreversible pulpitis with full pulpotomy. Santos et. al. showed that the short-term preoperative pulp inflammation did not jeopardize the outcome of pulpotomy [2]. Zinc oxide eugenol (ZOE), despite reported direct application toxicity, remains a prevalent pulpotomy liner in pediatric dentistry, credited for its sedative and palliative properties in alleviating pulpal pain [3]. In primary molar pulpotomies, diverse agents like ferric sulfate for hemostasis, formocresol (FC) and glutaraldehyde for fixation, corticosteroids for anti-inflammation, sodium hypochlorite as an antibacterial agent, and antibiotics like Ledermix for bacterial control are applied beneath ZOE as a barrier to shield the dental pulp. Recent prospective studies indicated a 65% success rate for ZOE over 24 months, notably lower than alternative pulpotomy materials namely MTA and generated controversy over its medicament preference [4].

Hyaluronic acid (HA), an oligosaccharide characterized by the repetition of β-1,4-*D*-glucuronic acid and β-1,3-*N*-acetyl-*D*-glucosamine disaccharide units, is abundant in connective and epithelial tissues. As a prominent component of the extracellular matrix, this mucopolysaccharide significantly contributes to cell proliferation and migration [5]. The wide range of molecular weights lends HA diverse elastic properties, rendering it suitable for various medical applications [6]. HA plays a crucial role in cell proliferation, migration, angiogenesis, wound healing, and tissue regeneration, with its concentration and molecular weight influencing physiological tissue properties [7]. This biopolymer structure acts as a protective barrier, a lubricant with viscoelastic properties in synovial fluid, and a filler material, inducing revascularization and enhancing regenerative capability in soft tissue [8–11].

Dental pulp, rich in glycosaminoglycan, demonstrates the contribution of HA to the dentin matrix and the initial developmental stages of dental pulp [12, 13]. Several in vitro and animal studies investigating the effect of hyaluronic acid on dental pulp reveal its positive impact. In a 1995 study comparing the effects of pulp capping with HA and calcium hydroxide on rat molar teeth, HA provided a conducive environment for reparative dentin formation through mesenchymal cell differentiation during dental pulp healing [8]. Numerous trials aimed at supporting the regenerative properties of pulpotomy materials were conducted [14, 15].

This study’s objective was to enhance the quality of ZOE by incorporating different concentrations of HA and assessing its impact on rat dental pulp tissue within the root canal. This investigation initially examined the physical properties, including hardness and compressive strength, of two commercially available ZOE materials mixed with hyaluronic acids at various concentrations in vitro. Subsequently, their histopathological effects on rat molar teeth were compared histologically in vivo.

## Materials and methods

Before conducting the animal experiment, an in vitro study was conducted, testing various concentrations of hyaluronic acid added to two different ZOE materials.

### In vitro experiment

#### Materials

Two different commercially available ZOE cements: IRM® (Dentsply Sirona, Charlotte, NC, USA) and Kalzinol® (Dentsply Sirona, Charlotte, NC, USA) were chosen for the assessment of prepared materials.

All HA preparations were carried out in Yildiz Technical University Faculty of Chemistry and Metallurgy Biotechnology Laboratory 1 day before the in vitro experiments. HA concentrations of 0.5%, 1%, and 3% by weight were prepared using commercial HA (Beijing Wisapple Biotech Co., LTD, Beijing, China) with a molecular weight of 1.6 MDa, obtained from bacterial sources and meeting medical injection-grade standards.

In addition, two commercially available HA products, Gengigel Teething® (Ricerfarma, Milano, Italy) containing 0.54% HA and Gengigel Forte® (Ricerfarma, Milano, Italy) 0.8% HA, by weight were included in the study.

#### Preparation of test samples

IRM® or Kalzinol® was mixed according to manufacturer’s directions. Immediately after mixing with powder and liquid ratio, equal amount of liquid HA at concentrations of 0.5%, 1%, or 3% as well as Gengigel Teething® or Gengigel Forte® was introduced into the mixture. The resulting materials were allowed to harden, and the prepared samples were subsequently immersed in distilled water at 37 ± 1 °C for 24 h. Prior to testing, the samples were equilibrated at 23 ± 1 °C for 15 ± 1 min. For the compressive strength test, 10 specimens, each with a diameter of 4 mm and a height of 6 mm, and for the Vickers hardness test, 5 specimens, each with a diameter of 10 mm and a height of 2 mm, were collected from each group. After the mixing of the samples was completed, they were placed in a cylindrical container within 1 min.

#### Assessment of physical properties

The physical properties of the materials were evaluated using two tests: compressive strength and Vickers hardness tests.

#### Compressive strength test

This test was conducted in accordance with ISO 3107:2011 standards, with results expressed in Megapascals (MPa). The dimensions of the ZOE pellets were recorded using a Vernier caliper (Mitutoyo 150 mm Digital Caliper, Mitutoyo Co., Kanagawa, Japan). The compressive strength was assessed at a crosshead speed of 1 mm/min and determined by dividing the maximum load the sample can withstand by its cross-sectional area [16, 17]. This experiment was replicated ten times for each of 12 groups.

#### Vickers hardness test

A diamond-tipped indenter was applied to the specimen surface under a 500 gf load for 20 s. Measurements were taken at five different points on each sample. Subsequently, a diamond-shaped mark was engraved onto the surface. The diagonal dimensions of this mark were measured using a microscope, and the VH value was calculated by dividing the load by the surface area of the indentation, as per the provided equation. [18, 19]. The test was conducted for 5 samples from each group.

### In vivo experiment

**Fig. 1:**
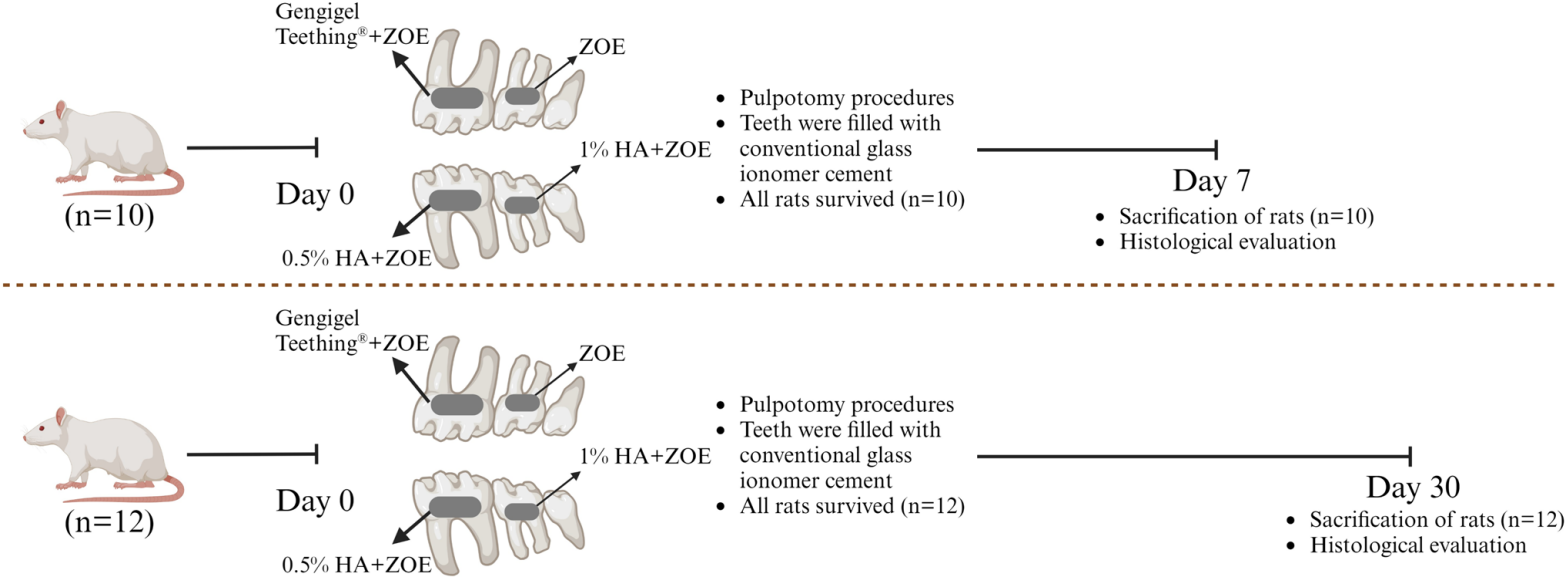
Study design of the experiment

Considering the outcomes of the in vitro experiment, Kalzinol® mixed with 0.5% or 1% concentrations of HA, or Gengigel Teething® gel were selected for the in vivo experiment. The design of the experimental study is *illustrated in *Fig. 1*.*

#### Animal ethics and sample collection

Ethical approval for the study was obtained from the Marmara University Local Ethics Committee for Animal Experiments under protocol code 30.2021mar. Based on a previous study, a sample size of 7 teeth per group attained a power of 95% and a significance level of 1% [20]. We used 10 teeth per sample for one group, and 12 teeth per sample for the other group. A total of 22 male Sprague Dawley rats, aged between 3–4 months and weighing 250–300 g, were provided from the Marmara University Laboratory Animal Application and Research Center for use in the experiments. In addition, the experimental procedures were performed complying with Guide for the Care and Use of Laboratory Animal, and all operations and post-operative care of the animals were carried out in Marmara University Laboratory Animal Application and Research Center. The rats were housed in a room with controlled light (12:12 h light–dark cycle) and temperature (22 ± 2 °C), with a constant relative humidity of 45–65%. They were accommodated in housing cages, with a maximum of four rats per cage. The animals were provided with a standard rat feed, and ad libitum access to water. After the operations, the rats were initially fed with commercially available soft food (Hero Baby, Istanbul, Turkey) for 2 days, and then provided ad libitum access to water and standard rat feed. The cages were regularly cleaned and maintained, with the bedding changed at least three times a week, the water and food of the animals were checked daily. Pre- and post-operative care of the animals was supervised by the responsible veterinarian at the Marmara University Experimental Animal Implementation and Research Center.

#### Pulpotomy procedure and sample division

On the day of the experiment, all procedures were conducted under strict sterile surgical conditions, adhering to aseptic, antiseptic, and sterilization protocols. Rats were anesthetized through intraperitoneal injection of 100 mg/kg ketamine-HCL (Ketalar, Eczacıbaşı, Istanbul, Turkey) and 10 mg/kg xylazine HCL (Rompun, Bayer, Germany). The depth of anesthesia and pain control in animals were monitored throughout the surgical procedure by assessing skin and toe pinch responses, as well as monitoring heart rate, respiratory rate, and other physiological parameters. Post-operation, analgesic (Paracetamol, orally at 100 mg/kg) was administered if pain symptoms were observed. Once adequate anesthesia was achieved, the animals were positioned supine on the operating table. Custom-designed retractors, tailored to allow easy access to the rats’ molar teeth, under artificial lighting, were inserted into the animals’ mouths, enabling visual identification of the target teeth. Pulp exposure was achieved in the designated molar teeth using a sterile round diamond burs (0.5–0.75 mm in diameter) (Shofu, San Marcos, CA, USA) in a high-speed handpiece. The coronal pulp was then extracted with the help of spoon excavator, followed by rinsing with sterile saline solution. Hemostasis was ensured using small cotton pellets.

The following procedures were carried out on each animals:

- Application of a mixture comprising 0.5% HA gel and the powder–liquid combination of ZOE on the first lower left molar. The mixture was applied immediately and adapted to the pulp surface.
- Application of a mixture comprising 1% HA gel and the powder–liquid combination of ZOE on the second lower left molar. The mixture was applied immediately and adapted to the pulp surface.
- Application of a mixture comprising Gengigel Teething® (Ricerfarma) gel and the powder–liquid combination of ZOE on the first upper left molar. The mixture was applied immediately and adapted to the pulp surface.
- Direct application of ZOE after achieving hemostasis in the exposed pulp on the second upper left molar as a control.

The preparation process for the material to be used in the pulpotomy proceeded as follows: The ZOE material was initially mixed according to the manufacturer’s instructions at a powder-to-liquid ratio of 5:1. Immediately thereafter, an equal amount of HA gel was added. To ensure precise measurement of liquid and HA, a micropipette (ISOLAB Laborgeräte GmbH, Eschau, Germany) was employed. The resulting mixture was then applied to cover the radicular pulp and allowed to harden for 10 min. The hydrogel was not applied directly; it was mixed with the material and then applied to the pulp.

Subsequently, conventional glass ionomer cement (GIC) (Kavitan™ Plus Radiopaque Glass Ionomer Cement, Pentron, Orange, CA, USA) was used to fill all treated teeth.

To reduce the operation duration, procedures were exclusively performed on the teeth on one side of the jaw, with intervention targeting the first and second molars due to their easier accessibility. A total of four teeth were intervened upon, comprising the maxillary and mandibular first and second molars on the left side of the rats. Identical materials were uniformly applied to these same teeth across all subjects.

#### Sample division and histological examination

Throughout the study period, all animals remained healthy. The animals were categorized into two groups based on the time period allocated for histological examination: 7-day group and 30-day group. After the stipulated time intervals, the animals were sacrificed with a high dose (200 mg/kg) of sodium pentobarbital (Pentothal, Abbott, Istanbul, Turkey) via intraperitoneal injection, and mandibular and maxillary samples were extracted for examination. These samples were sectioned to encompass the treated teeth. The mandibular and maxillary specimens were immersed in 10% neutral buffered formaldehyde (Sigma Aldrich, St. Louis, MO, USA) at room temperature and then transported to the Department of Pathology at Marmara University Faculty of Medicine.

Subsequently, they underwent the decalcification stage for 48 h using EDTA (ethylenediaminetetraacetic acid) disodium solution. The palates were then cut in a manner that allows visualization of the filling in the section and cassette-embedded. Following that, the tissues were processed through Thermo Scientific Shandon Excelsior AS tissue processor (Thermo Fisher Scientific, Waltham, MA, ABD), involving formaldehyde solution, alcohols (ethanol; 70%, 80%, 96%), xylene, and paraffin stages. The tissue processing procedure took 14 h. After the processing, the tissues were placed into appropriate base molds and solidified with melted paraffin at 65 °C. For each sample, a total of 20 sections, each measuring 5 μm in thickness, were mounted on slides and stained using hematoxylin and eosin (H&E) stain. For each sample, three slides were obtained, allowing visualization of the pulp. If deemed necessary, the number of sections was increased. All sections were reviewed for scoring, and an average of three repeated evaluations was conducted for optimal scoring. Furthermore, the histological specimens were examined using a light microscope (Olympus Optical Co., Tokyo, Japan). Magnifications of ×4, ×10, ×20 and ×40 were used during examination. All tissue samples were examined by a pathologist who was blinded to the treatment group allocations.

#### Histological assessment

The response of the pulp to treatment and the materials used was assessed according to the following criteria. These histological evaluations were determined and graded according to Sharma et al*.*, Lopes et al*.*, and Cengiz et al*.* [20–22]:

- 1. Formation of hard tissue: A “present” score was assigned for notable hard tissue deposition or complete dentin bridge formation near the exposed area, while an “absent” score was given in its absence.
  Score 0: present.
  Score 1: absent.
- 2. Canal obliteration and intracanal calcifications: Radicular tissues were assessed, and canal obliteration and intracanal calcifications were noted as present or absent.
  Score 0: present.
  Score 1: absent.
- 3. Continuity of the odontoblastic layer: The continuity of odontoblast cells was evaluated through sections and coded as present or absent.
  Score 0: present.
  Score 1: absent.
- 4. Pulp necrosis: According to the severity of tissue disorganization in the pulp, four scoring evaluations were conducted between vital and necrosis scores.
  Score 0: vital.
  Score 1: mild.
  Score 2: severe.
  Score 3: total.
- 5. Inflammation: Inflammation was scored based on the intensity of inflammatory changes and their distribution from the exposed area to the radicular zone as follows: absent, mild to moderate, and severe.
  Score 0: absent.
  Score 1: mild to moderate.
  Score 2: severe.
  Score 3: total.

### Statistical analysis

The normality of the data was assessed using the Shapiro–Wilk test, and a two-way analysis of variance was conducted to evaluate the effects of two different factors on the normally distributed compression and hardness variables. Significant interaction effects were assessed using the LSD multiple comparison test. The two-way analysis of variance (ANOVA) and LSD test used in our in vitro experiments are parametric tests.

For our in vivo experiment, relationships between categorical variables were tested using the Chi-square test which is a nonparametric test due to the absence of normal distribution assumption. SPSS Windows 22 software was employed for the analyses and a *P* < 0.05 was considered statistically significant.

## Results

### In vitro experiment

#### Compressive strength testing

**Fig. 2:**
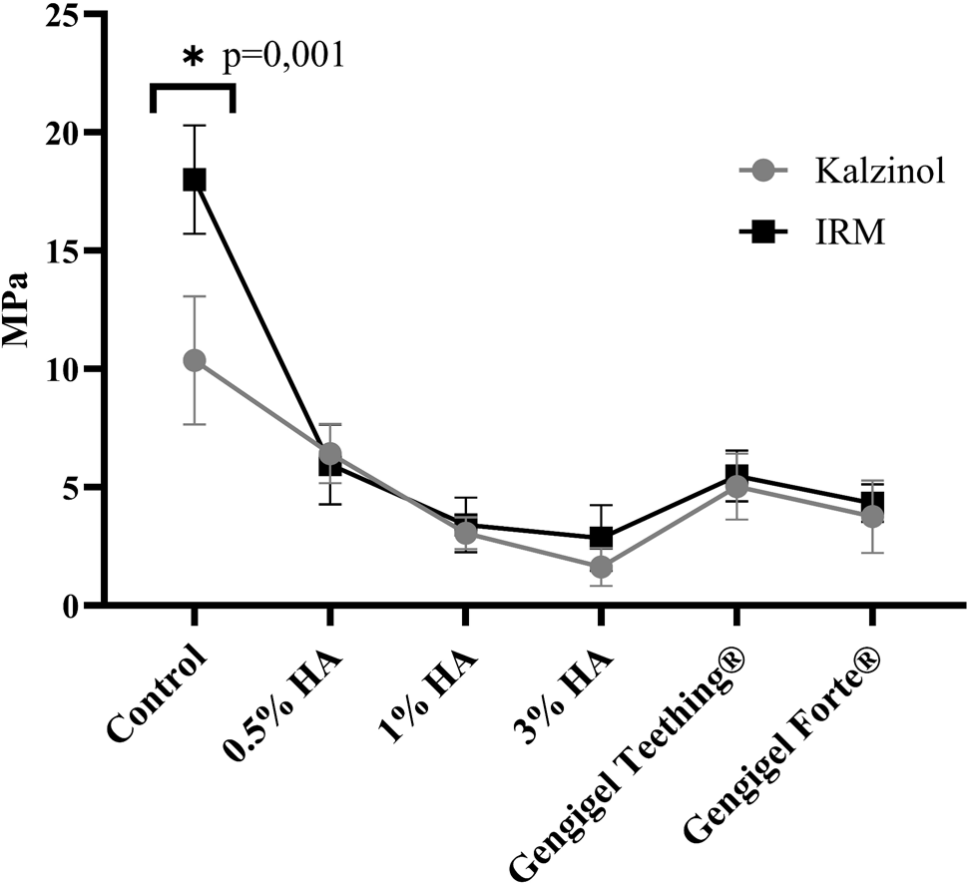
Average compressive strength values of the groups (MPa) and relationship of compressive strength test significance values between IRM® and Kalzinol® groups. * sign represents that there is a statistically significant difference between the control groups

The compressive strength test was repeated in 10 samples for each group. There was a statistically significant difference in compressive strength values between IRM® and Kalzinol® (*P* = 0.001). The compressive strength test indicated that elevating the concentration of HA in the mixture led to a reduction in the compressive strength value. Nonetheless, no significant difference was observed between the compressive strength values of HA-added IRM® and Kalzinol® materials. Mixtures with 0.5% HA, 1% HA, 3% HA, Gengigel Teething®, and Gengigel Forte® reduced the compressive strength of the IRM® by 66.01%, 81.17%, 84.73%, 69.37%, and 76.79%, and the compressive strength of the Kalzinol® by 37.98%, 70.43%, 84.27%, 51.45%, and 63.63%, respectively (Fig. 2).

The mixtures that exhibited average values exceeding 5 MPa meeting the standards were 0.5% HA-added, and Gengigel Teething®-added groups.

#### Vickers hardness testing

**Fig. 3:**
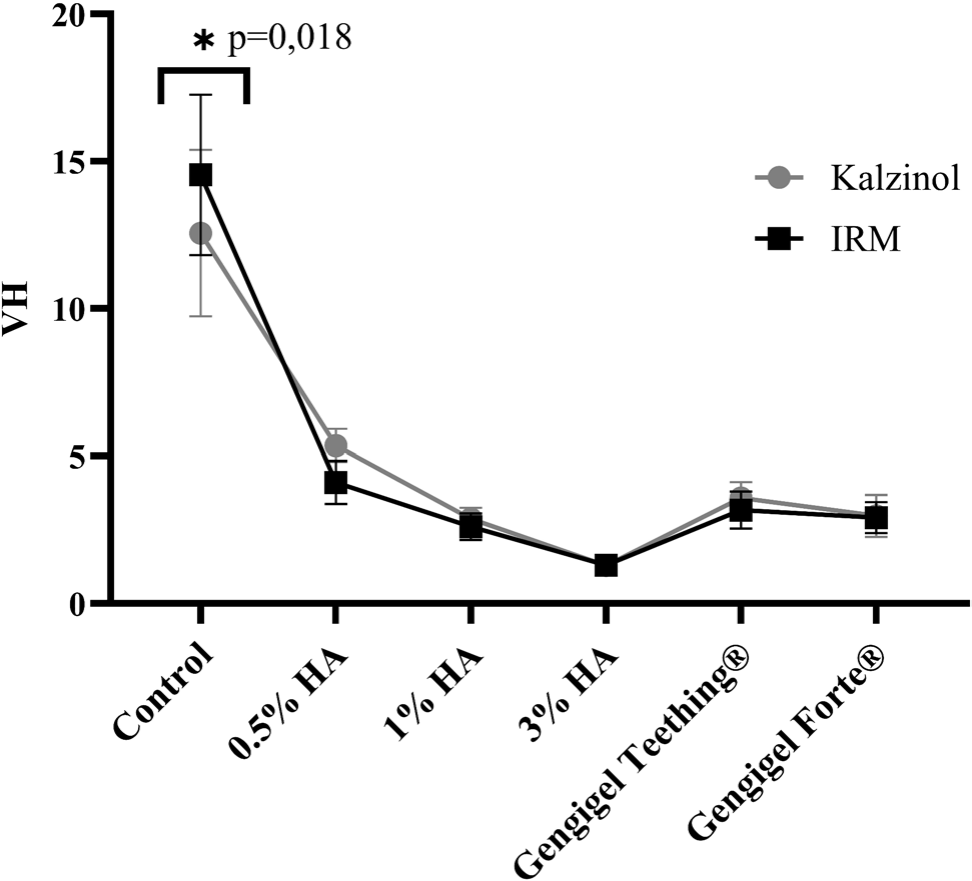
Average Vickers hardness (VH) values of the groups and relationship of VH test significance values between IRM® and Kalzinol® groups. * sign represents that there is a statistically significant difference between the control groups

The Vickers hardness test was repeated in 5 samples for each group. There was a statistically significant difference in Vickers hardness values between IRM® and Kalzinol® materials (*P* = 0.018). Vickers hardness test showed that the hardness value decreased with increasing HA concentration. However, there was no significant difference between the hardness values of HA-added IRM® and Kalzinol® materials. Mixtures prepared with 0.5% HA, 1% HA, 3% HA, Gengigel Teething®, and Gengigel Forte® reduced the hardness of the IRM® by 71.74%, 82.08%, 91.07%, 78.12%, and 79.92% and the hardness of the Kalzinol® by 57.32%, 77.07%, 89.76%, 71.79%, and 76.17%, respectively (Fig. 3).

### In vivo experiment

#### 7-day group

Odontoblastic layer continuity was seen in 90%, 70%, 70%, and 60% of teeth treated with 0.5% HA-, 1% HA-, Gengigel Teething®-added ZOE, and control ZOE, respectively. However, there was no statistically significant difference between the groups (*P* = 0.358). Both the 0.5% HA- and 1% HA-added ZOE groups exhibited mild to moderate inflammation in 50% of samples, with the remaining 50% showing severe inflammation. Teeth treated with Gengigel Teething®-added ZOE had 30% with no inflammation, 20% with moderate, and 50% with severe inflammation. Similarly, teeth treated with ZOE had only 10% no inflammation. There was no statistically significant difference between the groups (*P* = 0.218).

**Fig. 4:**
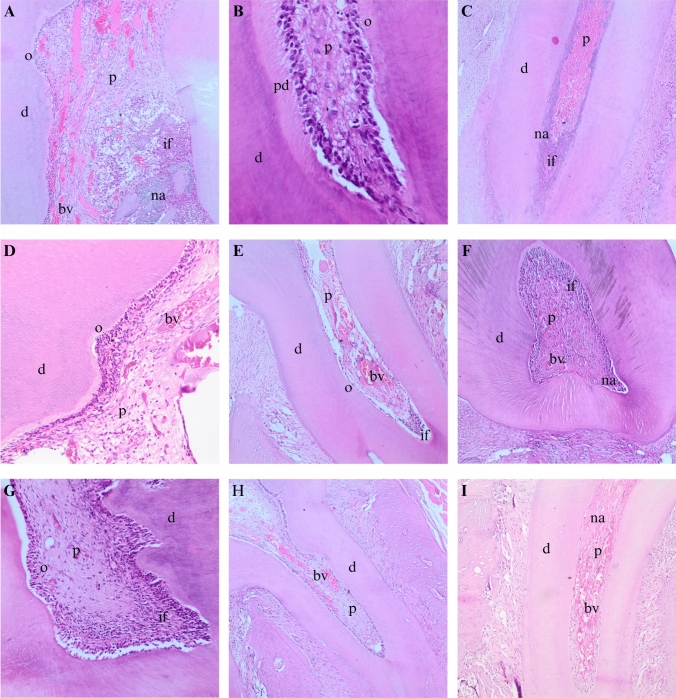
**a** 1 week after the procedure performed with 0.5% HA-containing ZOE, vital pulp (H&E, × 20); **b** 1 month after the procedure performed with 0.5% HA-containing ZOE, continuity of the odontoblast layer (H&E, × 10); **c** 1 month after the procedure performed with 0.5% HA-containing ZOE, partial-severe necrosis (H&E, × 10); **d** 1 week after the procedure performed with Gengigel Teething®-containing ZOE, vital pulp (H&E, × 20); **e** 1 month after the procedure performed with Gengigel Teething®-containing ZOE, continuity of the odontoblast layer (H&E, × 10); **f** 1 month after the procedure performed with Gengigel Teething®-containing ZOE, partial-severe necrosis (H&E, × 10); **g** 1 month after the procedure performed with ZOE alone, continuity of the odontoblast layer (H&E, × 20); **h** 1 week after the procedure performed with only ZOE, vital pulp (H&E, × 10); **i** 1 month after the procedure performed with only ZOE, partial-severe necrosis (H&E, × 10); *d* dentin, *p* pulp, *o* odontoblast, *na* necrosis area, *if* inflammation infiltration area, *bv* blood vessels, *pd* predentin

A statistically significant difference was found in the comparison of pulp vitality among the groups (*P* = 0.001). Following the pulpotomy procedure with 0.5% HA-added ZOE 50% of the teeth remained vital (Fig. 4a), 40% partially mild, and 10% as severe necrosis. In the 1% HA-added group, 60% were recorded as partially mild, and 40% as partially severe. Notably, no cases of necrosis were observed in either group. After the use of Gengigel Teething® gel and ZOE, 60% remained vital (Fig. 4d), 30% were severe, and 10% were necrotic. In the control group, 10% were vital (Fig. 4h), 70% were mild, 10% were severe, and 10% were necrotic (Table 1).

**Table 1:** Histopathological examination of the 7-day group

Parameter	0.5% HA-added ZOE	1% HA-added ZOE	Gengigel teething®-added ZOE	ZOE only	*P* value
Odontoblastic layer continuity					
Present	9 (90)	6 (60)	7 (70)	6 (60)	0.358
Absent	1 (10)	4 (40)	3 (30)	4 (40)	
Pulp necrosis					
Vital	5 (50)	0 (0)	6 (60)	1 (10)	0.001*
Mild	4 (40)	6 (60)	0 (0)	7 (70)	
Severe	1 (10)	4 (40)	3 (30)	1 (10)	
Total	0 (0)	0 (0)	1 (10)	1 (10)	
Inflammation					
Absent	0 (0)	0 (0)	3 (30)	1 (10)	0.218
Mild to moderate	5 (50)	5 (50)	2 (20)	4 (40)	
Severe	5 (50)	5 (50)	5 (50)	5 (50)	

*HA* hyaluronic acid, *ZOE* zinc oxide eugenol

^*^Significant at *P* < *0.05* level, Chi-square test

#### 30-day group

Hard tissue formation was seen in 91.7%, 80%, 81.8%, and 54.5% of the teeth treated with 0.5% HA-, 1% HA-, Gengigel Teething®-added groups, and control groups, respectively. However, no statistically significant difference was found among the groups (*P* = 0.197). Intracanal calcification was observed in 75%, 70%, 81.8%, and 30% of the teeth treated with 0.5% HA-, 1% HA-, Gengigel Teething®-added, and control groups respectively. No statistically significant difference was observed in the canal obliteration and intracanal calcifications parameter (*P* = 0.064). While 1% HA-added and control groups had total inflammation, 25% and 18.6% of partial inflammation were seen in 0.5%HA-added and Gengigel Teething®-added groups, respectively. Despite these variations, no statistically significant difference was noted among the groups (*P* = 0.194).

Odontoblast layer continuity was observed in 25%, 63.6%, and 20% of 0.5% HA-added, Gengigel Teething®-added, and control groups (Fig. 4b, e, g). A statistically significant difference was found among the groups (*P* = 0.005). Moreover, the pulp vitality was found to be significantly different among the groups (*P* = 0.018). 66.7%, 80%, 63.6%, and 90% had total necrosis in 0.5% HA-, 1% HA-, Gengigel Teething®-added, and control groups, while severe necrosis was noted in the remaining specimens (Fig. 4c, f, i) (Table 2).

**Table 2:** Histopathological examination of 30-day group

Parameter	0.5% HA-added ZOE	1% HA-added ZOE	Gengigel teething®-added ZOE	ZOE only	*P* value
Hard tissue formation					
Present	11 (91.7)	8 (80)	9 (81.8)	6 (54.5)	0.197
Absent	1 (8.3)	2 (20)	2 (18.2)	5 (45.5)	
Canal obliteration and intracanal calcifications					
Present	9 (75)	7 (70)	9 (81.8)	3 (30)	0.064
Absent	3 (25)	3 (30)	2 (18.2)	7 (70)	
Odontoblastic layer continuity					
Present	3 (25)	0 (0)	7 (63.6)	2 (20)	0.005*
Absent	9 (75)	10 (100)	4 (36.4)	8 (80)	
Pulp necrosis					
Severe	4 (33.3)	2 (20)	7 (63.6)	1 (10)	0.018*
Total	8 (66.7)	8 (80)	4 (36.4)	9 (90)	
Inflammation					
Mild to moderate	2 (16.7)	0 (0)	2 (18.2)	0 (0)	0.194
Severe	1 (8.3)	0 (0)	0 (0)	0 (0)	
Total	9 (75)	11 (100)	9 (81.8)	11 (100)	

*HA* hyaluronic acid, *ZOE* zinc oxide eugenol

^*^Significant at *P* < *0.05* level, Chi-square test

## Discussion

Ongoing research persists in advancing endodontic dental materials for pediatric dentistry, specifically aiming to identify an optimal material for primary dentition. Although HA has not been incorporated into any commercially available endodontic material to date, it holds potential as a candidate. However, its utilization may necessitate supplementation with other dental materials due to inherent structural characteristics [23, 24]. In this study, various concentrations of HA were introduced into ZOE materials. A comprehensive analysis was then conducted to assess the effects of applying these HA-added ZOE materials to the dental pulp of rats. The study revealed histological success, particularly at lower concentration.

The rheological examination of HA-added materials is commonly undertaken due to their predominant application in gel form. In addition, researchers have explored mixed stiff HA/collagen and HA/gelatin scaffolds [9, 25]. There is no study evaluating the mechanical properties of a dental material containing hyaluronic acid, used as a base, similar to our research.

### In vitro compressive strength and hardness

Per ISO 3107:2011, Type 2 ZOE materials must display a minimum compressive strength of 5 MPa. Manappallil suggested acceptable values, recommending 6 MPa to 28 MPa for Type 1 temporary cements and 5 MPa to 55 MPa for Type 2 bases and temporary restorations [26]. In a study by Karimy et al*.*, commercially available ZOE showed compressive strengths ranging from 5.67 to 8.5 MPa, and Anderson et al*.* reported variations from 4.1 to 21.61 MPa [27, 28]. This study evaluated compressive strength values of 0.5% to 3% concentrations of HA added to two commercially available ZOE products. Both 0.5% HA-added IRM® or Kalzinol® and Gengigel Teething® (0.54% HA)-added IRM® or Kalzinol® met these standards, suggesting that concentrations exceeding 0.5% HA did not compromise ZOE material compressive strength.

In a study evaluating the mechanical properties of ZOE mixtures with varying sizes and shapes of zinc oxide particles, samples with a small average particle size, homogeneous distribution, and an oxygen-rich surface demonstrated the highest compressive strength and hardness. This performance was attributed to the large particle surface area, facilitating enhanced interfacial bonding [29]. Materials incorporating HA exhibited diverse viscoelastic properties, showing behavior ranging from dilute to entangled solutions based on HA concentration and molecular weight. Recent attempts have been made to enhance the mechanical stability of hydrogels [30]. In this study, a significant reduction in hardness was observed in both HA-added IRM® and Kalzinol® materials, potentially impacting material durability in clinical settings. Further studies are required to delve deeper into this aspect.

### In vivo experiment results

After in vitro experiments, 3% HA-added ZOE samples were excluded from the animal experiment group due to poor results in compressive strength and Vickers hardness tests. However, 1% HA-added ZOE was retained for experimentation. Overall, significant differences were observed in pulp vitality within the 7-day groups and in odontoblast layer continuity and pulp vitality within the 30-day groups.

Many studies have aimed to assess the impact of different materials on rat pulp histologically. Sharma et al*.* focused on the reparative effects of keratin hydrogel in rat molars after partial pulpotomy [21]. Several histological evaluation criteria, such as inflammatory cell response, necrosis, vitality, and mineralization, aligned with those in our study. Lopes et al*.* conducted a comparative analysis of MTA and ferric sulfate in pulpotomy, with MTA demonstrating superior histological features, higher interleukin-6 expression, and similar inflammatory cell counts [22]. Our study similarly investigated the inflammatory response, revealing no significant differences between the groups. Cengiz et al*.* evaluated sodium alendronate as an alternative for pulpotomy, showing similar pulp vitality preservation and hard tissue formation as calcium hydroxide, with no significant difference in inflammatory response and vascularization [20]. In our study, we considered the lateral exposure site for the evaluation of hard tissue formation, the roots for assessing canal obliteration, and the entire tooth in cross-sectional evaluations for other histological assessments.

There are numerous studies assessing the effects of diverse application of HA on dental pulp in animals. Bogovic’s investigation into the direct use of HA in pulp capping suggested a higher potential for reparative dentin formation in the HA group with superior cell viability and minimal apoptosis and necrosis [31]. Umemura et al*.* concluded that HA induces odontoblastic differentiation without affecting cell proliferation, utilizing high-molecular-weight HA for induced mineralization [32]. Chrepa et al*.* explored cell scaffolds containing Restylane (HA) or Matrigel, with Restylane demonstrating successful outcomes in terms of survival, mineralization, and odontoblastic activity [33]. Chen et al*.* evaluated high-molecular-weight HAs for regenerative pulp therapies, showcasing their potential as pulp capping and filling materials [23].

On the other hand, Ildes et al*.* examined the efficacy of HA as a pulpotomy medicament in human primary molars, comparing it with FC and ferric sulfate treatments over a 12-month period, both clinically and radiographically. Their findings revealed no statistically significant differences among the groups [11]. Similarly, Mahfouz et al*.* investigated a 1:1 mixture of high-molecular-weight HA gel and ZOE cement for pulpotomy in primary molars, finding its success comparable to FC pulpotomy after 12 months [34]. These in vivo short-term studies collectively emphasized the versatile and promising role of HA in various aspects of dental research, ranging from reparative dentin formation to odontoblastic differentiation and regenerative endodontic treatments.

In our study, the 7-day group showed that odontoblastic layer continuity and inflammation were lowest in 0.5% HA groups among the treatment groups, without any statistically significant difference. The 30-day group results showed that these parameters were still higher especially in Gengigel teething®-added group which has similar 0.5% HA content.

**Fig. 5:**
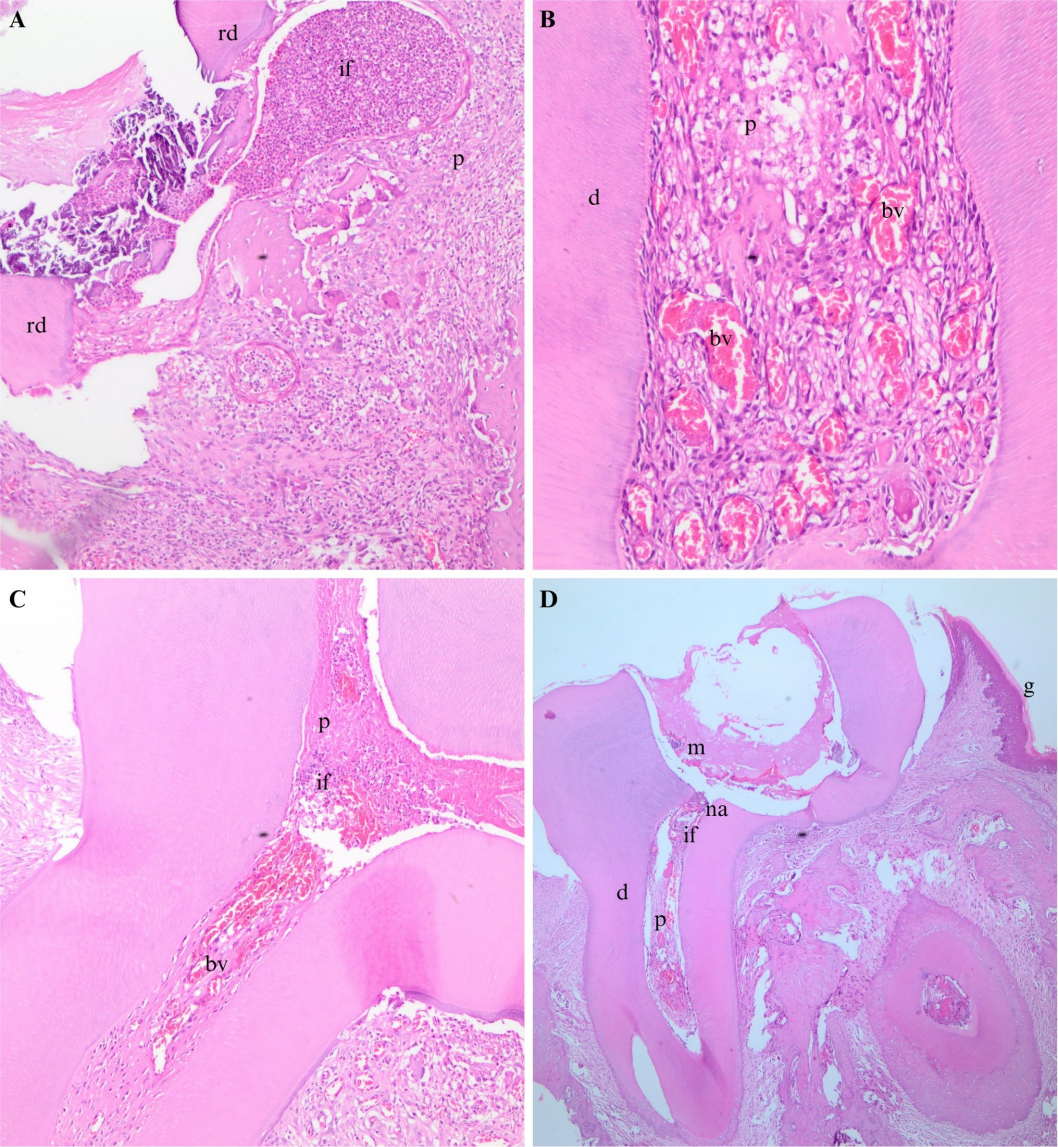
Congestion and inflammation examples in 7-day group:** a** (H&E, × 20), **b** (H&E, × 20), **c** (H&E, × 10), **d** (H&E, × 4); *d* dentin, *p* pulp, *na* necrosis area, *if* inflammation infiltration area, *bv* blood vessels, *rd* reparative dentin, *m* material, *g* gingiva

In the 7-day group, a noticeable reduction and rarefaction of odontoblasts were evident. This impact was observed in both coronal and root odontoblasts. Specifically, within the coronal pulp of the 7-day group, discernible bacterial clusters, signs of inflammation, congestion, and the presence of micro-abscesses were noted (Fig. 5a–d). Notably, pulp vitality exhibited a significant difference, underscoring the impact of the applied formulations on short-term outcomes. Furthermore, necrosis was identified in the coronal area of this group, leading to the loss of odontoblasts within the necrotic regions.

The 30-day group revealed distinctions in hard tissue formation, intracanal calcification, odontoblast layer continuity, and pulp vitality among the treatment groups. The combination of Gengigel Teething® with ZOE demonstrated superior outcomes in certain parameters, such as odontoblast layer continuity, suggesting a potential positive influence of the combination approach. However, the lack of statistical significance in some parameters implies the need for further investigation and potentially larger sample sizes to draw robust conclusions. In contrast, Palma et al. examined the effectiveness of lyophilized hydrogel HA scaffolds for dental pulp regeneration in immature dog teeth. The experimental groups comprised either blood clotting or two different formulations of a chitosan hydrogel as scaffolds. Despite the initial expectations, the addition of chitosan scaffolds to blood in regenerative procedures in dogs did not yield significant improvements in the formation of new mineralized tissues along the root canal walls or provide histological evidence of the regeneration of a pulp-dentin complex [35].

**Fig. 6:**
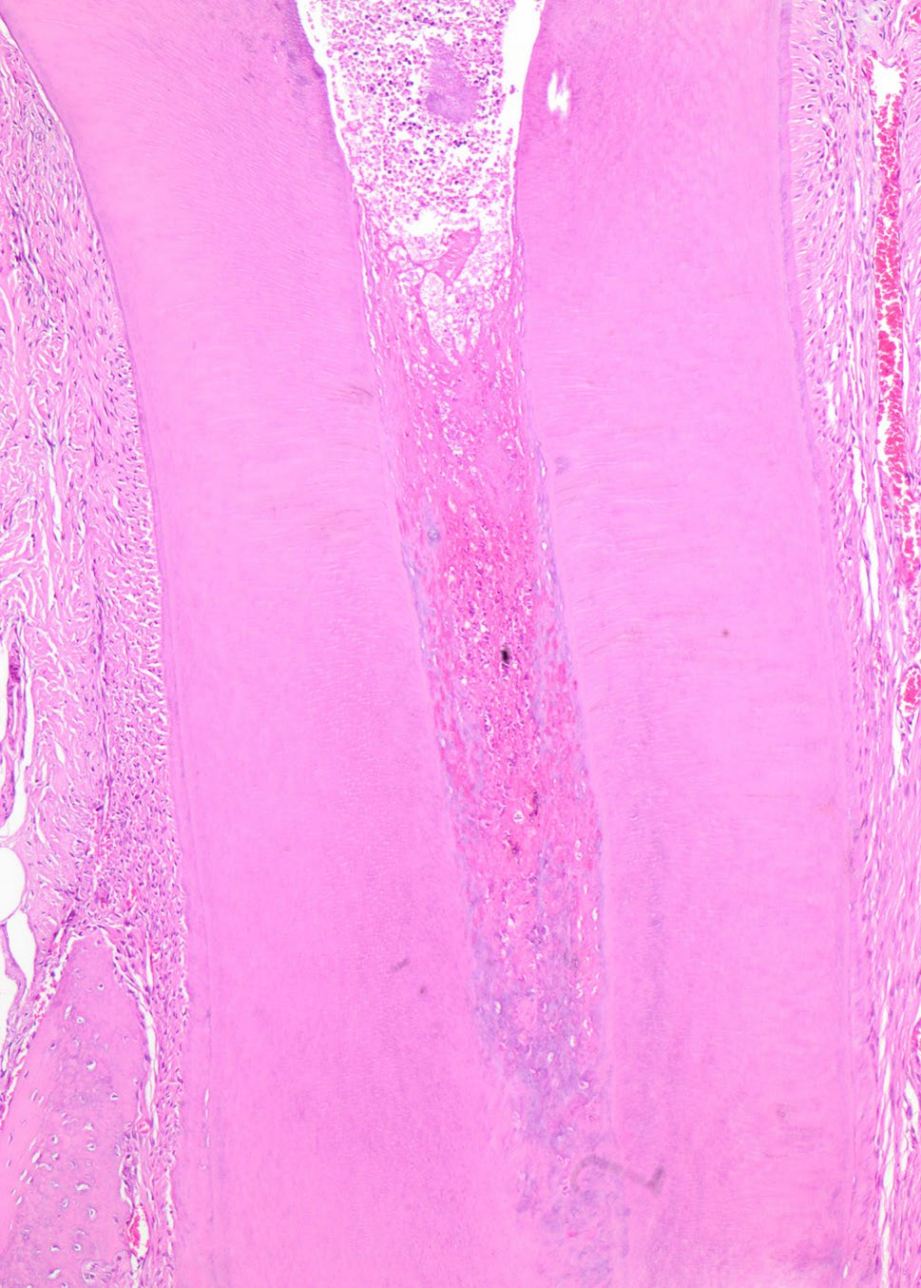
Obliteration in 30-day group (H&E, × 10)

In the 30-day group, calcifications and pulp obliterations were observed (Fig. 6). Notably, the infection and necrosis detected in the coronal region extended to the root in certain cases. In addition, in some teeth, osteoclastic regenerative changes were noted, possibly attributed to a foreign body reaction related to perforation during the experimental procedure. These features were possibly due to the accelerated biological response in rat teeth as compared to humans [6, 36, 37].

Reinforced zinc oxide eugenol cements remain popular due to their cost-effectiveness compared to calcium silicate-based materials. Therefore, considering its clinical preference and continued relevance, we selected ZOE for the in vivo animal experiment section of our research. However, direct application onto dental pulp has been linked to various toxic effects, including chronic inflammation and an increased risk of internal resorption [1].

For the past 50 years, studies have focused on tissue reactions following direct pulp capping, pulpotomy, and pulp exposure in rat teeth [8, 9, 20–22, 36–38]. Numerous studies have indicated that histological healing of the pulp in rat teeth treated with calcium hydroxide in pulp capping treatments is similar to that observed in human teeth [8, 39]. Therefore, researchers suggest that studies on rat teeth could be utilized as an appropriate model.

During the study, there were several factors complicating work on rat molars. Factors such as narrow mouth opening of rats, very small dimensions of teeth and pulps, and the anatomical position of teeth being far back in the oral cavity (long diastema) caused technical difficulties when working on rat teeth. Limitations of this study include the restricted number of animals and teeth used during the operation. We believe that increasing the number of rats could render some of the findings statistically significant. Conversely, the limited number of molar teeth in each rat prevented us from investigating different HA-added groups for each rat. We chose to study rat molars, despite the higher regenerative potential of incisors in rats. Molar teeth were preferred to observe realistic histological responses, as they possess a structure containing pulp tissue that closely resembles human molars in terms of anatomy, physiology, histology, and biology. However, it remains challenging to make direct comparisons with primary molars in humans, which have distinct physiological resorptive features.

While rapid inflammatory responses in rats raise questions about the optimal timing for realistic pulpal evaluation, future studies can adjust evaluation times and concentrations. Control groups, such as calcium hydroxide or MTA, may be employed for comparison. In addition, incorporating various counterstains or immunohistochemical markers can enhance the assessment of odontogenic, angiogenic, and neurogenic potential.

Furthermore, the success of the performed treatment is influenced not only by the base material placed on the pulp but also by the shear bond between the subsequently applied restoration and the base material. Studies have examined the shear bond of materials such as MTA and Biodentine with glass ionomer cement applied over them [40]. In the in vitro and in vivo experiments, alongside ZOE, groups such as MTA and Biodentine could have been selected, and the bond between them and GIC used as the restoration could have been evaluated. In vitro study limitations include challenges in determining the released amount of HA using chromatographic methods, measuring material setting times and pH levels, and employing imaging techniques to identify variations in surface morphology.

In previous studies, the direct effect of hyaluronic acid on the pulp was investigated solely through gel form preparations applied in pulp capping and pulpotomy treatments, with additional filling materials applied over the hyaluronic acid. A pulpotomy material containing hyaluronic acid has not been developed or tried. In this regard, our study bears the distinction of being a first. Confirmation of the designed material’s effectiveness not only eliminates a step in the multi-stage pulpotomy treatment, reducing time, but also suggests that HA, a tissue-friendly substance, could replace other agents, reducing toxicity risks. The data from this study may contribute to the development of a new pulpotomy material in the future.

In conclusion, our exploration for a more optimal concentration of HA added to ZOE and the results from in vivo experiments have shed light on the intricate dynamics of ZOE material incorporating HA. Significantly, our study stood out as the first inquiry into the influence of ZOE material containing HA on dental pulp, providing valuable insights into the short- and medium-term effects of various pulpotomy approaches. This contribution advances the broader investigation of dental pulp regeneration and treatment efficacy. The outcomes of our study suggest that HA holds promise as a material for pulpotomy and regenerative endodontic treatments. These findings indicate that the use of such materials in dental procedures, especially in pediatric cases, should be approached with careful consideration. Further research is essential to better understand the long-term clinical implications of these materials in dental applications.

## Data Availability

The data that support the findings of this study are available from the corresponding author upon reasonable request.
